# Involvement of fatty acid synthase in dengue virus infection

**DOI:** 10.1186/s12985-017-0685-9

**Published:** 2017-02-13

**Authors:** Natthida Tongluan, Suwipa Ramphan, Phitchayapak Wintachai, Janthima Jaresitthikunchai, Sarawut Khongwichit, Nitwara Wikan, Supoth Rajakam, Sutee Yoksan, Nuttaporn Wongsiriroj, Sittiruk Roytrakul, Duncan R. Smith

**Affiliations:** 1 Molecular Pathology Laboratory, Institute of Molecular Biosciences, Mahidol University, Salaya Campus, 25/25 Phuttamonthon Sai 4, Salaya, Nakhon Pathom, Bangkok 73170 Thailand; 2 National Center for Genetic Engineering and Biotechnology (BIOTEC), National Science and Technology Development Agency, Pathum Thani, 12120 Thailand; 3 Center for Emerging and Neglected Infectious Diseases, Mahidol University, Nakhon Pathom, Bangkok 73170 Thailand

**Keywords:** Dengue, Lipid, non-structural protein 3, Fatty acid synthase, Orlistat

## Abstract

**Background:**

The mosquito transmitted *Dengue virus* (DENV) remains a significant public health problem in many tropical and subtropical countries. Increasing evidence has suggested that during the infection process cellular lipids play important roles at several stages of the replication cycle. This study sought to characterize the changes in lipid metabolism gene expression and investigated the role of one enzyme, fatty acid synthase, in DENV infection.

**Methods:**

Transcriptional profiles of genes associated with lipid metabolism were evaluated by real-time PCR after infection of different cell lines (HepG2 and HEK293T/17) and with different DENVs (laboratory adapted and low passage). Expression profiles of genes were evaluated by western blotting. A critical lipid metabolism protein, fatty acid synthase was down-regulated through siRNA and inhibited with orlistat and the effect on DENV infection determined by flow cytometry, plaque assay, western blotting and confocal microscopy.

**Results:**

The results showed alterations of gene transcription and expression were seen in genes variously associated with lipogenesis, lipolysis and fatty acid β-oxidation during DENV infection. Interference of fatty acid synthase with either siRNA or orlistat had marked effects on virus production, with orlistat having an EC_50_ value of 10.07 μM at 24 h post infection. However, non-structural protein expression was largely unaffected.

**Conclusions:**

While drug treatment reduced virus titer by up to 3Log10, no significant effect on DENV non-structural protein expression was observed, suggesting that fatty acid synthase acts through an effect on virion formation.

**Electronic supplementary material:**

The online version of this article (doi:10.1186/s12985-017-0685-9) contains supplementary material, which is available to authorized users.

## Background

Each year there are believed to be nearly 400 million new *Dengue virus* (DENV) infections in tropical and sub-tropical countries worldwide, of which some 100 million show some form of symptom [1]. The mosquito transmitted DENV infects humans after the bite of an infected female Aedes mosquito, and where symptoms occur these can range from a mild flu-like illness to a severe life threatening syndrome primarily characterized by plasma leakage [2]. DENV is largely maintained in an urban transmission cycle, with the anthropophilic *Aedes aegypti* being the primary vector [3]. DENV consists of four closely related viruses, DENV 1 to 4, and while infection with one virus results in the induction of a robust protective immune response against the infecting virus [4], only transient or no immunity is offered against infection with a heterotypic virus, and repeated infections with heterotypic viruses can occur [5]. In many cases, second infections are associated with a more severe presentation as a consequence of interplay between the host immune response raised by the first infection and the second heterotypic virus [6].

The virus is believed to initially replicate in skin resident dendritic cells, after which transmission of the virus around the body can result in the involvement of a wide range of cell types including monocytes and macrophages [7], megakaryocytes [8], erythroid precursor cells [9], liver cells [10] and endothelial cells [11]. Internalization of the virus to a host cell is believed to occur by endocytosis of the virus via clathrin coated pits after receptor binding [12], although alternate pathways have been proposed [13, 14]. The DENV genome, a single stranded positive sense RNA molecule encoding a single open reading frame is directly translated as a single polypeptide that undergoes processing by viral and host proteases to generate the three structural proteins (envelope (E), pre-membrane (prM) and capsid proteins) and the seven non structural proteins (NS1, NS2A, NS2B, NS3, NS4A, NS4B and NS5) that form the replication complex [2]. The replication complex directs the production of the full length positive sense genome and the newly synthesized genomic RNA is packaged by the capsid protein forming a nucleocapsid complex [15]. The nucleocapsid buds from the ER and becomes enveloped by a lipid membrane in which are embedded the newly synthesized E and prM proteins. As a consequence of this process, nearly 20% of the weight of the dengue virion is lipid [16].

Despite the virus particle having significant lipid content [16], this aspect of DENV pathobiology is only recently being explored. A comprehensive analysis undertaken in insect cells showed that infection resulted in extensive remodeling of the lipid profile, in particular with respect to alterations in the levels of lipids that were associated with altering membrane bilayer curvature or permeability [17]. In mammalian cells it is know that virus entry is associated with the expression of receptors associated with lipid rafts [18], and that cholesterol required for infection [19]. DENV infection results in significant remodeling of membranes to provide structures for the replication complex [20], as well as possibly shielding the replication complex from the host cellular innate immune system [21]. Given the extensive membrane re-modeling in mammalian cells (as with insect cells), it is likely that the mammalian cell lipid profile is also re-engineered to support DENV replication. It is known that β-oxidation is increased in DENV infected cells [22], and it has been proposed that the main consequence of the activation of autophagy in DENV infection [23, 24] is to increase β-oxidation to provide energy for DENV replication [22]. Consistent with this, the re-localization of fatty acid synthase (FASN; a critical rate limiting enzyme in lipid biosynthesis) to the replication complex by interaction with DENV NS3 has been reported [25, 26].

This study sought to determine whether lipid metabolism associated gene expression is altered in mammalian cells upon DENV infection, as well as to determine whether targeting proteins involved in lipid metabolism affects the DENV replication cycle. The study showed that the expression of lipid associated genes is altered during DENV infection, and that FASN is a suitable target for therapeutic intervention.

## Methods

### Cell lines and viruses

HEK293T/17 (Human Embryonic Kidney) and HepG2 (Human hepatocellular liver carcinoma) cells were maintained in Dulbecco’s modified eagle’s medium (DMEM) supplemented with 10% fetal bovine serum (FBS) and 100 units of penicillin/streptomycin per ml. LLC-MK2 (Rhesus monkey kidney epithelial) cells were grown in the same medium containing 5% FBS and 100 units/ml of penicillin/streptomycin. All cultures were grown at 37 °C in a 5% CO_2_ humidified incubator. DENV 2 (strain 16681) DENV 4 (strain 1036) or DENV-4DHF (GeneBank accession number KM519592) were as described previously [27]. Viruses were propagated in C6/36 cells and virus titers were determined by standard plaque assay as described previously [28]. Virus infection with was undertaken as described previously [28] in the presence or absence of orlistat (Sigma-Aldrich, St Louis, MO).

### Oil red O staining

Mock infected or DENV 2 infected HEK293T/17 or HepG2 cells were grown under standard conditions in six well plates for 24 h following which the cells were fixed by the addition of 1 ml of 10% formalin followed by 1 ml of 60% isopropanol for 10 min. The cells were stained with Oil Red O (Sigma-Aldrich), washed with distilled water 4 times before oil red O was eluted from the cells by the addition of 1 ml of 100% isopropanol for 15 min and the absorbance of the solution measured by spectrophotometry at 490 nm. Experiments were undertaken independently in triplicate. For visualization of oil red droplets, the experiment was repeated with cells grown on glass coverslips (without elution of Oil Red O), followed by observation under bright field of a Carl Zeiss LSM800.

### Real time PCR

HEK293T/17 or HepG2 cells were mock infected or infected at m.o.i 5 (DENV 2) or at m.o.i 20 (DENV 4 and DENV-4DHF) and incubated for 24 h under standard conditions, after which time total RNA was extracted using Tri-Reagent (Molecular Research Center, Cincinnati, OH) and treated with DNase for 1 h followed by re-extraction with Tri-Reagent. 1 μg of total RNA was reverse transcribed to cDNA using Improm-п™ reverse transcriptase (Promega, Madison, WI) and oligo (dT) (Bio Basic, Inc.). Quantitative real time PCR was undertaken using the KAPA SYBR FAST qPCR Kit 2X Master MIX (Kapa Biosystems Inc, Wilmington, MA) in a Mastercycler ep realplex real-time PCR system. Amplification using gene specific primers (Additional file 1: Table S1) was carried out at 95 °C for an initial 3 min period followed by 40 cycles of denaturation at 95 °C for 10s, annealing at 60 °C for 30s and extension at 72 °C for 20s. Transcripts examined included those for fatty acid synthase (FASN), adipose triglyceride lipase (ATGL) gene, peroxisome proliferator-activated receptor alpha (PPARα), diglyceride acyltransferase 1 (DGAT1), acetyl-CoA carboxylase alpha 1 (ACC1), Fatty acyl CoA oxidase (AOX), Carnitine palmitoyltransferase I (CPT1), Medium-chain acyl-CoA dehydrogenase (MCAD), sterol regulatory element-binding protein-1c (SREBP1c), stearoyl-CoA desaturase 1 (SCD1) and actin. Experiments were performed independently in triplicate.

### siRNA gene silencing

siRNA templates were designed from target sites on the human fatty acid synthase gene (GenBank accession number NM_004104) and the green fluorescent protein (GFP; GenBank accession number U50974) using the online tool from Ambion, Austin, TX (http://www.ambion.com/techlib/misc/siRNA_finder.html). The sequences were subjected to siRNA template design to generate DNA oligonucleotides sequences for the Silencer siRNA Construction Kit (Ambion). Templates for siRNA generation were as follows:

- siFASN1: 1275-AACGTGGGCATCAACTCCTTT-1295
- siFASN2: 3191-AACTGGGTGAGCTTCATGGAC-3211
- siFASN3: 5477-AAGAACGTGACATTCCACGGG-5497
- siFASN4: 7112-AACAGCCTCTTCCTGTTCGAC-7132
- siGFP: 295-AAAGATGACGGGAACTACAAG-315

### siRNA transfection and infection

HEK293T/17 cells were maintained in antibiotic free medium and transfected using lipofectamine RNAiMAX (Invitrogen,Carlsbad, CA) by reverse transfection following the manufacturers protocol using 250 pmol siRNA and 2.5 μl of lipofectamine RNAiMAX. The cells were cultured under standard conditions for 24 h. At 24 h post-transfection, transfected and mock transfected cells were infected with DENV 2 m.o.i 5 for 2 h. After that, uninternalized viruses were inactivated by an acid glycine wash as described previously [28]. At 24 and 48 h post transfection, supernatants and cells were collected for analysis by plaque assay and flow cytometry, respectively.

### Cytotoxicity assessment

Cytotoxicity of orlistat was evaluated using the Vybrant® MTT Cell Proliferation Assay Kit according to the manufacturers’ recommendation. Cytotoxicity was evaluated at 24 and 36 h at 10, 50 and 100 μM. In addition, cell morphology was assessed under an inverted light microscope.

### Flow cytometry

Infected and mock infected cells were collected by centrifugation and blocked with 10% normal goat serum and incubated on ice for 30 min following which the cells were fixed with 4% paraformaldehyde and incubated at room temperature in the dark for 20 min and then permeabilized with 0.2% saponin for 10 min. The cells were washed with 1% BSA in PBS and centrifuged at 6,000 g for 5 min. Cells were resuspended and incubated overnight at 4 °C with an anti-dengue virus complex mouse monoclonal antibody (Merck Millipore, Billerica, MA). Subsequently cells were incubated with a FITC conjugated goat anti-mouse IgG antibody (Thermo Scientific Inc, Rockford, IL). Finally, the cells were resuspended in PBS and were analyzed on a BD FACalibur cytometer (Becton Dickinson, BD Biosciences, San Jose, CA, USA) and data analyzed using CELLQuestTM software.

### Immunofluorescence and confocal imaging

Immunofluorescence was undertaken essentially as described previously [24]. HEK293T/17 cells grown on glass cover slips were mock infected or infected with DENV 2 m.o.i 5 for 2 h at 4 °C with or without treatment of the cells with compound as indicated. After the appropriate time period cells were washed twice washed with PBS for 5 min and were subsequently permeabilized with 0.3% TritonX-100 in PBS for 10 min and then washed twice with 0.03% TritonX-100 in PBS. Cells were subsequently incubated with a 1:50 dilution of a rabbit polyclonal anti-dengue NS1 antibody (Thermo Scientific Inc.) at 4 °C overnight, after which cells were washed three times with 0.03% TritonX-100 in PBS for 5 min each time, followed by incubation with a 1:100 dilution of a rhodamine red X-conjugated goat anti-rabbit IgG antibody (Jackson ImmunoResearch Laboratories) and a 1:500 dilution of DAPI (Molecular Probes) for 1 h. Finally, the cells were washed with 0.03% TritonX-100 in PBS and the cover slips were mounted onto glass slides using Prolong® Gold antifade reagent (Invitrogen). Signals were observed under an Olympus FluoView 1000 confocal microscope (Olympus Corporation, Shinjuku-ku, Tokyo, Japan) equipped with Olympus FluoView Software v. 1.6. For colocalization analysis, the same protocol was used, but the primary antibody incubation was undertaken with a 1:50 dilution of a rabbit monoclonal anti-DENV NS3 protein antibody (Thermo Fisher Scientific) and a 1:50 dilution of a mouse monoclonal anti-fatty acid synthase (FASN) antibody (Santa Cruz Biotechnology Inc.). Secondary antibodies were a Rhodamine red- conjugated goat anti-rabbit IgG (Thermo Fisher Scientific) and a Alexa 488-conjugated donkey anti-mouse IgG (Molecular Probes). Cells were additionally stained with DAPI (Moleclar Probes).

### Western blot analysis

Mock infected or DENV 2 infected HEK293T/17 cells, either untreated or treated with compound as appropriate were lysed with RIPA buffer (1% Nonidet P-40, 0.5% sodium deoxycholate, 0.1% SDS in PBS) and the lysates were sonicated twice for 5 min at 4 °C. The cell lysates were then centrifuged at 10,000 x g for 10 min and supernatants were collected. Equal amounts of protein (80 μg) were separated on 10% SDS-polyacrylamide gels and were transferred to nitrocellulose membranes using a Trans-Blot electrophoretic transfer cell (Bio-Rad Laboratories, Richmond, CA). The membranes were blocked with 5% skim milk in TBS for 2 h at room temperature before incubation with appropriate primary antibodies, namely a 1:500 dilution of a mouse monoclonal pan specific anti-dengue virus antibody (Santa Cruz Biotechnology), a 1:1,000 dilution of a rabbit monoclonal anti-dengue NS3 antibody (Thermo Fisher Scientific), a 1:1,000 dilution of a mouse monoclonal anti-fatty acid synthase (FASN) antibody (sc-55580; Santa Cruz Biotechnology Inc.), a 1:1,000 dilution of a rabbit anti-ACC monoclonal antibody (#3662; Cell Signaling Technology Inc., Danvers, MA), a 1:2,000 dilution of a rabbit anti-PPARγ polyclonal antibody (sc-7196; Santa Cruz Biotechnology Inc.) or a 1:3,000 dilution of a goat anti-actin polyclonal antibody (sc-1616; Santa Cruz Biotechnology Inc.) before incubation with a 1:8000 dilution of a horseradish peroxidase (HRP) conjugated rabbit anti-mouse IgG polyclonal antibody (Thermo Fisher Scientific), a 1:10,000 dilution of a HRP conjugated goat anti-rabbit IgG (Pierce Rockford, IL) or a 1:8,000 dilution of HRP conjugated rabbit anti-goat IgG polyclonal antibody (31402; Pierce, Rockford, IL). Signals were detected using the Amersham ECL plus Western Blotting Detection Reagents (GE Healthcare) and autoradiography film.

### Statistical analysis

Data were analyzed using the GraphPad Prism program, statistical analysis of significance were undertaken by paired-sample T Tests using SPSS statistic 17.0 (SPSS Inc., Chicago, IL) with value of p < 0.05 for significance. CC_50_ and EC_50_ values were calculated using the freeware ED50plus (v1.0) software as described previously [29].

## Results

### Lipids in DENV infection

**Fig. 1:**
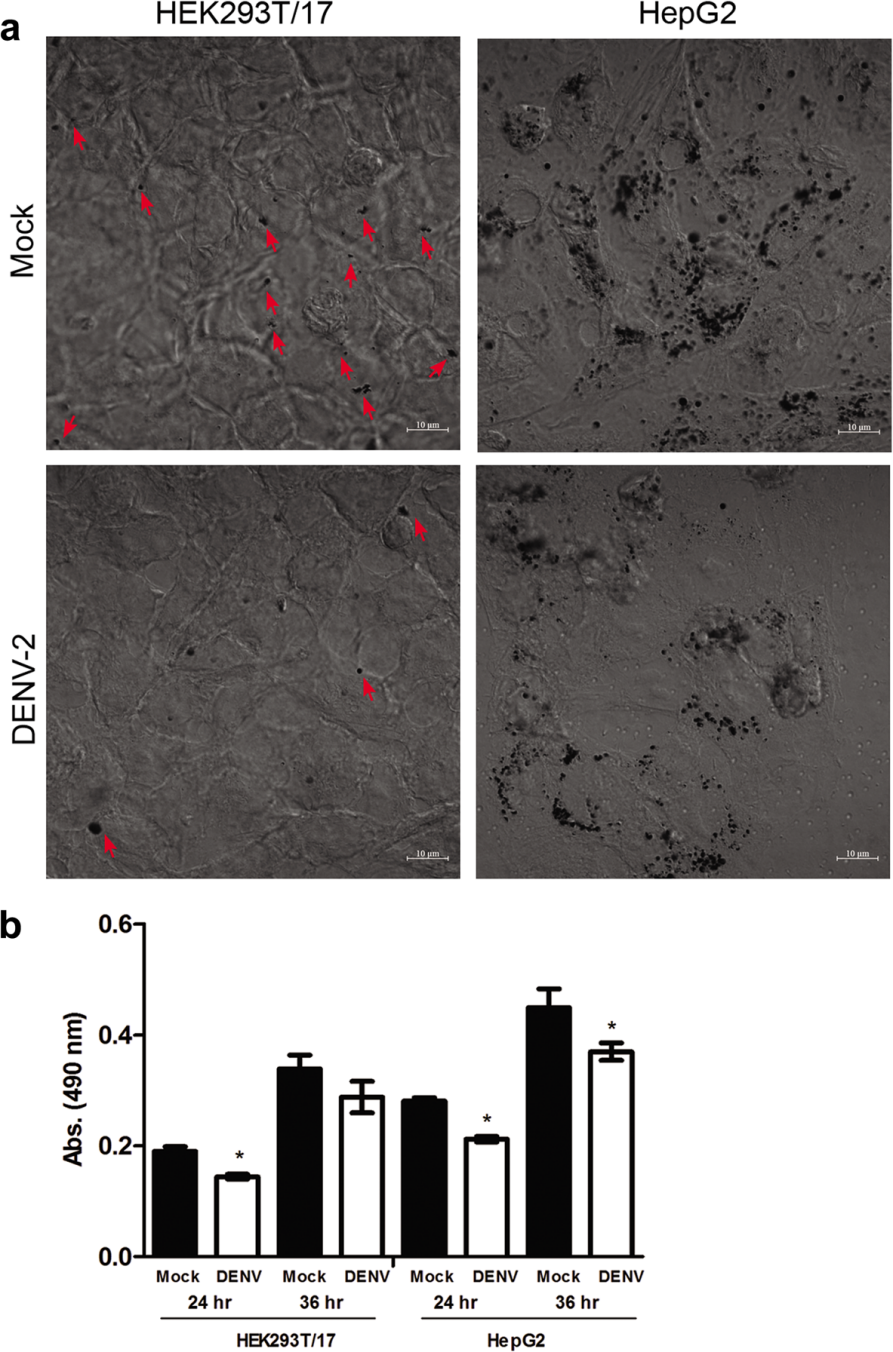
Observation and quantification of neutral lipids by Oil *red* O staining. DENV 2 infected and mock infected (**a**) HEK293T/17 cells and (**b**) HepG2 cells cultured on glass microscope cover slips for 24 h were stained with Oil *red* O before observation under bright field of a Carl Zeiss LSM800 microscope. Oil droplets show as black circles. The smaller droplets in HEK293T/17 cells are arrowed. The staining was repeated on cells grown in six well plates for 24 and 36 h and Oil *red* O was subsequently eluted and quantified by spectrophotometry. Experiment was undertaken independently in triplicate. Bars show mean +/−SD (*; *p* value <0.05)

To begin to investigate the involvement of lipids in DENV infection we first focused on neutral triglycerides. HEK293T/17 and HepG2 cells were therefore either mock infected or infected with DENV 2, and at 24 h post infection examined by light microscopy after staining with Oil Red O, a diazo dye that stains neutral triglycerides. While neither of the examined cell types examined showed large lipid droplets, both cell types showed results consistent with the reduction of lipid droplets as a consequence of infection (Fig. 1a, b) as established by others [22]. The reduction in neutral triglycerides was confirmed by elution of the dye, and spectrophotometric quantitation (Fig. 1c), although the difference between DENV infected at non-infected cells was lost in HEK293T/17 cells at 36 h.p.i.

### Evaluation of lipid pathway gene transcripts

To evaluate the mRNA expression of genes involved in lipid metabolism, the transcripts of genes mediating lipogenesis (fatty acid synthase (FASN), acetyl CoA carboxylase alpha (ACC1), sterol regulatory element-binding protein-1c (SREBP1c), diglyceride acyltransferase (DGAT) and stearoyl-CoA desaturase (SCD1)), lipolysis (peroxisome proliferator activated receptor (PPARα) and adipose triglyceride lipase (ATGL)) and fatty acid oxidation (fatty acyl CoA oxidase (AOX), medium-chain acyl-CoA dehydrogenase (MCAD) and carnitine palmitoyltransferase I (CPT1) were determined by quantitative real time PCR in mock infected and DENV 2 infected HEK293T/17 and HepG2 cells.

**Fig. 2:**
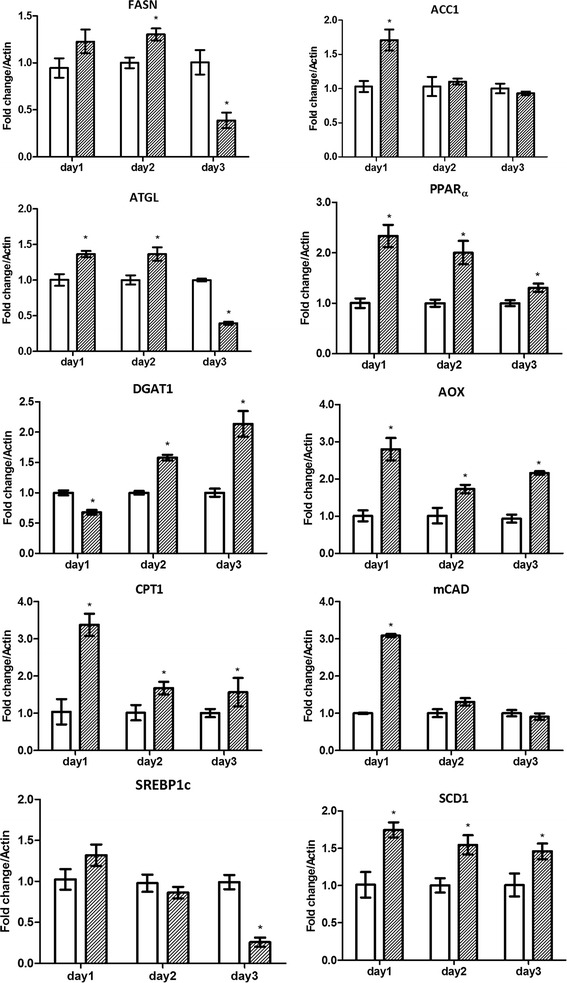
Profiles of lipid metabolism associated gene transcripts in response to DENV 2 infection of HEK293T/17 cells. HEK293T/17 cells were either infected (*grey bars*) with DENV 2 or mock infected (*white bars*) and the transcripts of fatty acid synthase (FASN), acetyl CoA carboxylase alpha (ACC1), adipose triglyceride lipase (ATGL), peroxisome proliferator activated receptor (PPARα), diglyceride acyltransferase (DGAT), fatty acyl CoA oxidase (AOX), carnitine palmitoyltransferase I (CPT1), medium-chain acyl-CoA dehydrogenase (MCAD), sterol regulatory element-binding protein-1c (SREBP1c) and stearoyl-CoA desaturase (SCD1) genes analyzed by real time PCR on days 1–3 p.i. Experiment was undertaken independently in triplicate. Bars show mean +/−SD (*; *p* value <0.05)

**Fig. 3:**
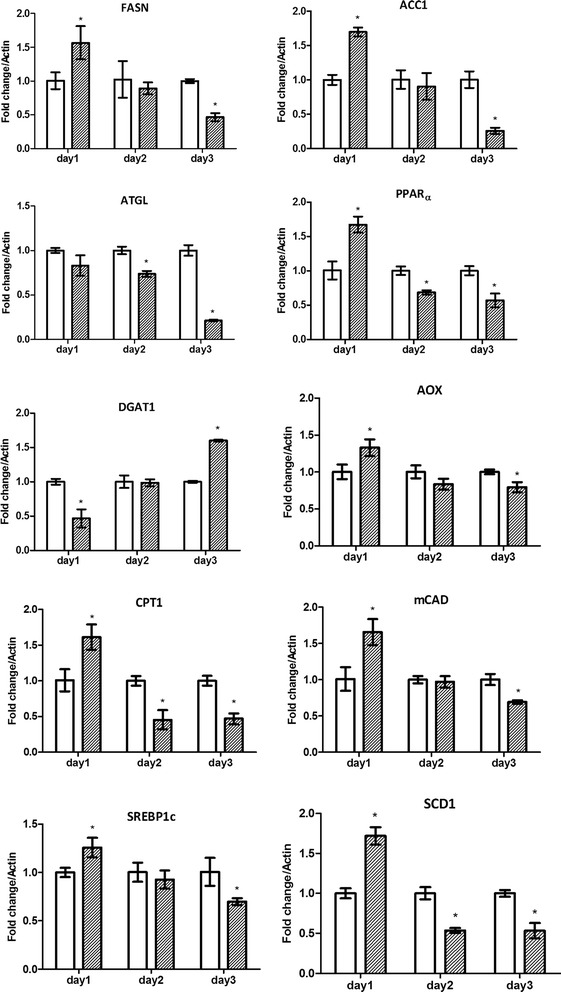
Profiles of lipid metabolism associated gene transcripts in response to DENV 2 infection of HepG2 cells. HepG2 cells were either infected (*grey bars*) with DENV 2 or mock infected (*white bars*) and the expression of fatty acid synthase (FASN), acetyl CoA carboxylase alpha (ACC1), adipose triglyceride lipase (ATGL), peroxisome proliferator activated receptor (PPARα), diglyceride acyltransferase (DGAT), fatty acyl CoA oxidase (AOX), carnitine palmitoyltransferase I (CPT1), medium-chain acyl-CoA dehydrogenase (MCAD), sterol regulatory element-binding protein-1c (SREBP1c) and stearoyl-CoA desaturase (SCD1) genes analyzed by real time PCR on days 1–3 p.i. Experiment was undertaken independently in triplicate. Bars show mean +/−SD (*; *p* value <0.05)

Results (Figs. 2 and 3) showed that the trend of gene transcription was broadly similar for genes in both cell lines, with transcripts significantly increasing at 24 h in DENV 2 infected cells as compared to mock infected cells, and levels of transcription generally decreasing thereafter. Notable exceptions to this trend were DGAT1, which in both cell lines showed a reduction in gene transcription at 24 h in infected cells (Figs. 2 and 3), and a significant increase in gene transcription by day 3 post-infection as compared to mock infected cells, as well as the lipogenesis genes FASN and SREBP1c in HEK293T/17 cells and the lipolysis gene ATGL in HepG2 cells (Fig. 2 and 3). For the majority of genes that showed an initial increase, all transcripts in HepG2 infected cells showed reduced levels by day 3 post infection (below the level of mock infected cells), while for HEK293T/17 cells, some transcripts still showed an increase over mock by day 3 (PPARα, AOX and SCD1), while others were either not different from mock at day 3 (ACC1, CPT1, MCAD) or significantly reduced as compared to mock infected cells (FASN, ATGL, SREBP1c). Combined however, the results (with some exceptions) were consistent with a broad initial up-regulation of lipid metabolism gene transcription, followed by a decline in gene transcription over the next 2 to 3 days post infection.

### Influence of serotype and passage history

**Fig. 4:**
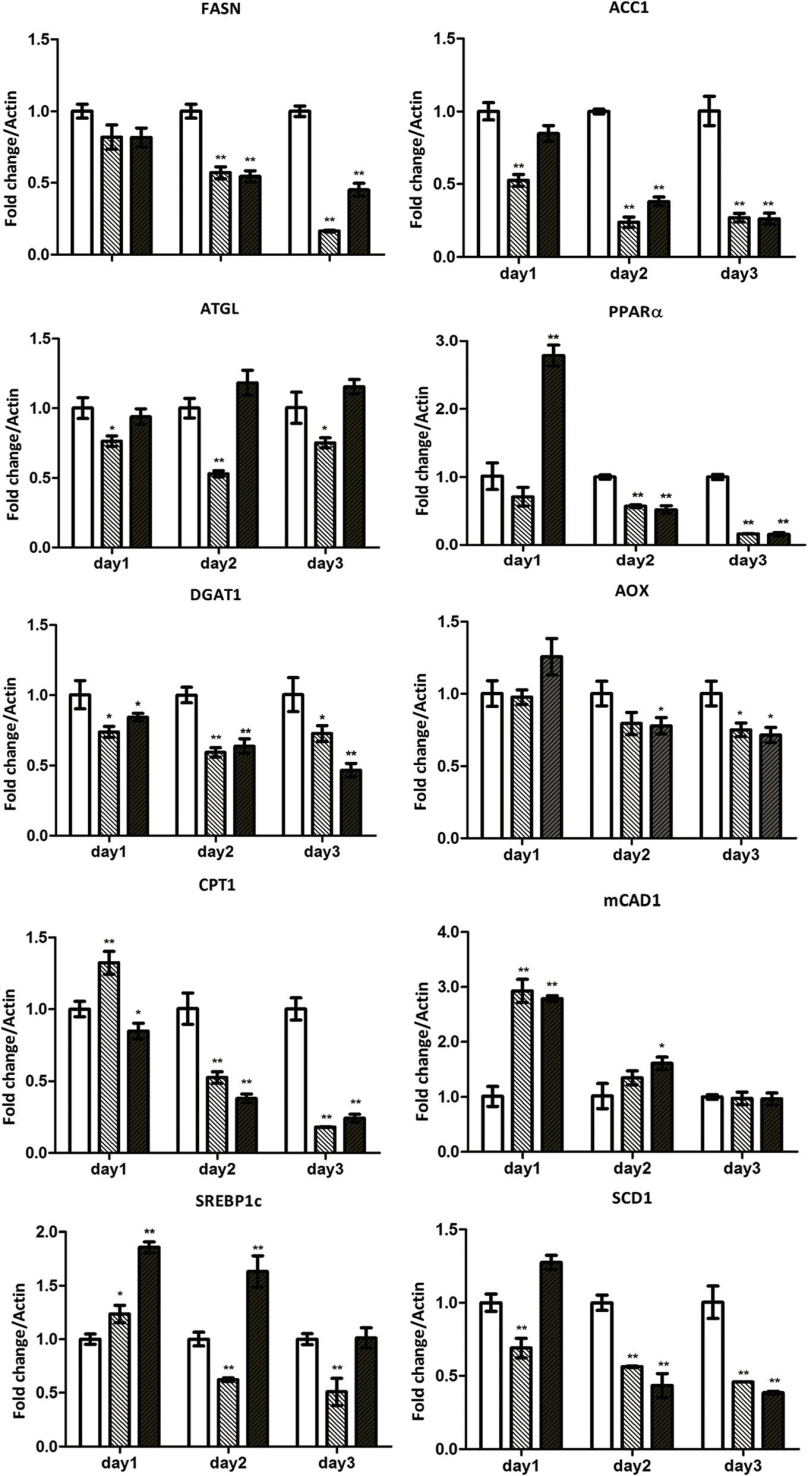
Profiles of lipid metabolism associated gene transcripts in response to infection of HEK293T/17 cells with DENV 4. HEK293T/17 cells were either infected with a laboratory adapted DENV-4LAB (*grey bars*) or a low passage DENV-4DHF (*black bars*) as previously described [27] or mock infected (*white bars*) and the expression of fatty acid synthase (FASN), acetyl CoA carboxylase alpha (ACC1), adipose triglyceride lipase (ATGL), peroxisome proliferator activated receptor (PPARα), diglyceride acyltransferase (DGAT), fatty acyl CoA oxidase (AOX), carnitine palmitoyltransferase I (CPT1), medium-chain acyl-CoA dehydrogenase (MCAD), sterol regulatory element-binding protein-1c (SREBP1c) and stearoyl-CoA desaturase (SCD1) genes analyzed by real time PCR on days 1–3 p.i. Experiment was undertaken independently in triplicate. Bars show mean +/−SD (*; *p* value <0.05)

The lipid gene transcription profile was repeated, this time using a laboratory adapted DENV-4LAB, and a low passage DENV 4 isolated from a dengue hemorrhagic fever patient (DENV-4DHF) [27], although only HEK293T/17 cells were investigated. Results (Fig. 4) again showed a degree of dysregulation of transcription of genes involved in the different aspects of lipid metabolism (lipogenesis, lipolysis and fatty acid oxidation), although there were marked differences from the changes seen in response to DENV 2 infection, and indeed, differences were observed between DENV-4LAB and DENV-4DHF, particularly on day 1 post infection. While the majority of genes showed down-regulation in transcription as compared to mock on days 2 and 3 post infection, discordant results were observed for three genes, namely ATGL, mCAD1 and SREBP1c (Fig. 4).

### Evaluation of lipid pathway protein expression

**Fig. 5:**
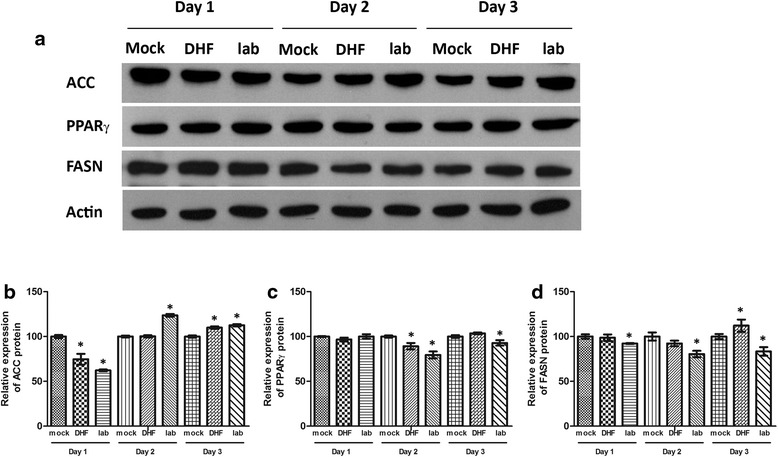
Expression of lipid metabolism associated proteins in response to infection of HEK293T/17 cells with DENV 4. HEK293T/17 cells were either infected with a laboratory adapted DENV-4LAB (lab) or a low passage DENV-4DHF (DHF) as previously described [27] or mock infected (Mock) and (**a**) the expression of acetyl CoA carboxylase alpha (ACC), peroxisome proliferator activated receptor (PPARγ) and fatty acid synthase (FASN) determined by western blotting together with actin. Experiments were undertaken independently in triplicate and signals quantitated (**b**). Bars show mean +/−SD (*; *p* value <0.05)

We further investigated changes in protein expression of genes involved in lipid metabolism, and levels of three proteins (ACC1, PPARγ and FASN) were determined by western blotting on days 1 to 3 post infection after infection with DENV-4LAB and DENV-4DHF or mock infection. Results (Fig. 5) showed some alterations of expression, although alterations in protein expression level were not as marked as alterations in gene expression.

### DENV 2 infection of FASN silenced HEK293T/17 cells

**Fig. 6:**
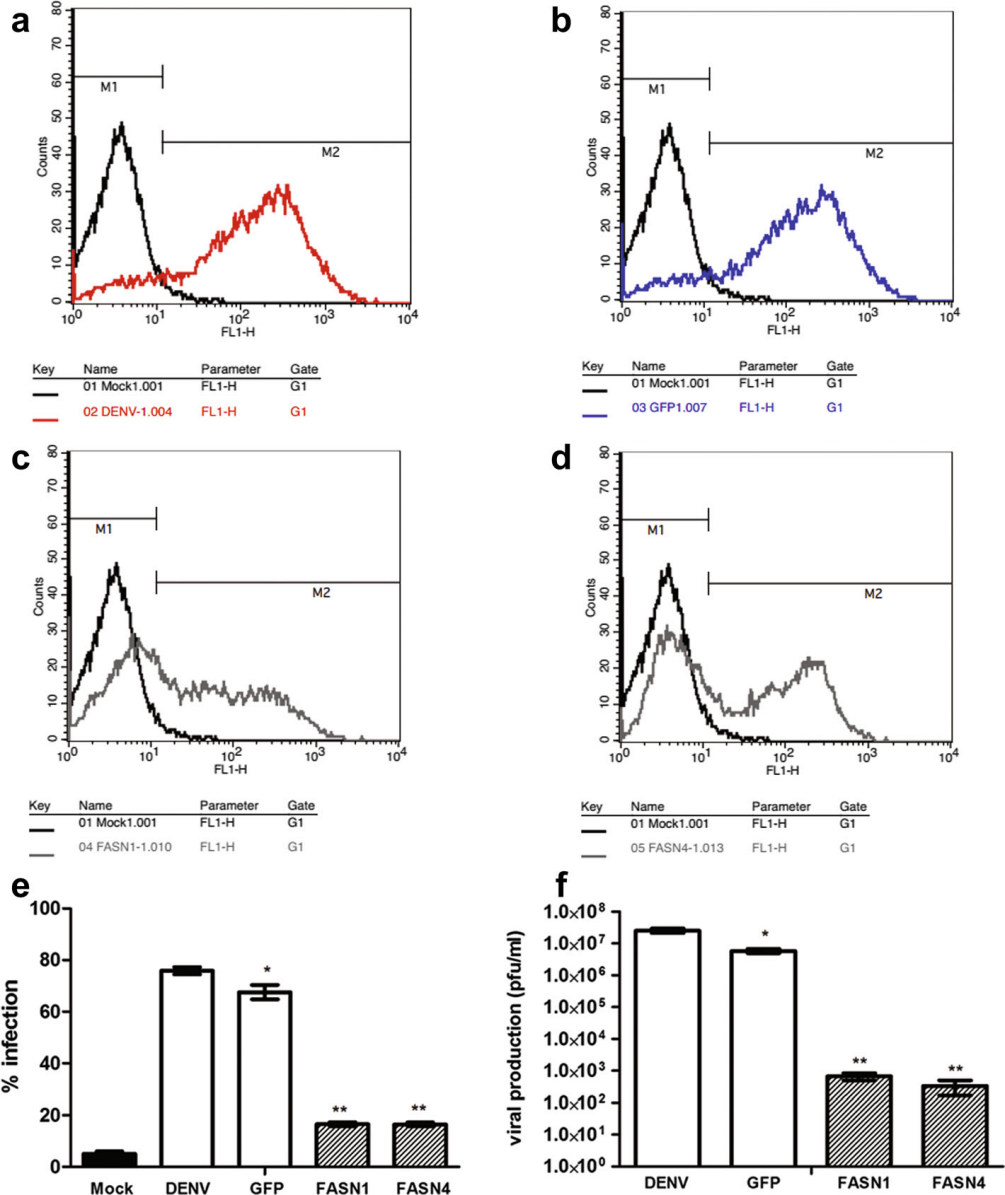
DENV 2 infection of HEK293T/17 cells with down-regulated fatty acid synthase (FASN). HEK293T/17 cells were treated with siRNAs targeted to FASN (FASN1 and FASN4) or a control siRNA (GFP) or not treated (DENV) and subsequently infected with DENV 2. At 24 h.p.i both percentage infection and virus titer were determined. Experiments were undertaken independently in triplicate, with duplicate plaque assay. (**a** to **d**) representative flow cytometry histograms with gating. M1: uninfected cell population, M2: infected cell population. *Black* line is mock infected (the same for all histograms), **a** *red*: DENV infected, **b** siGFP transfected and DENV infected, **c** siFASN1 transfected and DENV infected, **d** siFASN4 transfected and infected. **e** Bar chart showing M2 populations from three independent replicates and (**f**) bar chart showing results of plaque assay. Bars show mean +/−SD (*; *p* value <0.05)

The modulation of lipid metabolism associated gene expression during DENV infection suggests that this process might be a suitable anti-DENV target. To explore this FASN, a critical enzyme in lipogenesis [30] was down regulated through the use of siRNA. A panel of four different siRNAs (FASN1 - FASN4) was initially investigated for the ability to reduce FASN mRNA expression as assessed by real time PCR. Results showed that two siRNAs (FASN1 and FASN4) were able to largely abolish FASN expression on day 2 post transfection (Additional file 2: Figure S1), with no associated effects on cell viability (Additional file 2: Figure S2). Due to the very low expression detected by real time PCR for FASN1 and FASN4, the experiment was repeated independently (Additional file 2: Figure S3) and results again showed a dramatic reduction in expression with FASN1 but a more modest reduction for FASN4. To confirm the lack of transcript seen with FASN 1, amplification products for day 2 post transfection were additionally run on agarose gels (Additional file 2: Figure S3). A significant reduction in FASN protein expression after siRNA transfection was confirmed by western blotting (Additional file 2: Figure S4). Subsequently HEK293T/17 cells were transfected with siRNAs directed against FASN (FASN1 and FASN4) or against GFP, and on day 2 post transfection infected with DENV 2 in parallel with non-siRNA treated cells and mock infected cells. On day 1 post infection, cells were analyzed for infection by flow cytometry, and the supernatant examined for virus titer. Results showed that the percentage of infection was significantly reduced in FASN siRNA treated cells as compared to siGFP or cells not treated with siRNA (Fig. 6), and that virus titer was reduced by some 4Log10 in FASN siRNA treated cells as compared to siGFP or cells not treated with siRNA (Fig. 6).

### Evaluation of anti-FASN drug treatment to inhibit DENV infection

To evaluate the possibility of drug targeting towards lipid metabolism as an anti-DENV therapy, one drug, orlistat (tetrahydrolipstatin) was selected for evaluation. Cell cytotoxicity towards HEK293T/17 cells was evaluated using the MTT assay and cell morphology was additionally observed under an inverted light microscope. Results (Additional file 2: Figure S5) showed no cytotoxicity at 24 h post treatment over the range of concentrations tests (10 μM to 100 μM), and no discernible changes in morphology over the same time period (Additional file 2: Figure S6). However at 36 h post treatment, while the MTT assay showed no significant difference (Additional file 2: Figure S5), discernible morphological changes with orlistat treatment above 50 μM were seen (Additional file 2: Figure S6). The CC50 values were 1580 μM and 557 μM for orlistat at 24 and 36 h respectively. The possibility of this drug having a direct virucidal activity on DENV was additionally evaluated, and no loss of either cell infectivity or virus titer produced was observed after directly incubating DENV with the drug before infection (Additional file 2: Figure S7).

**Fig. 7:**
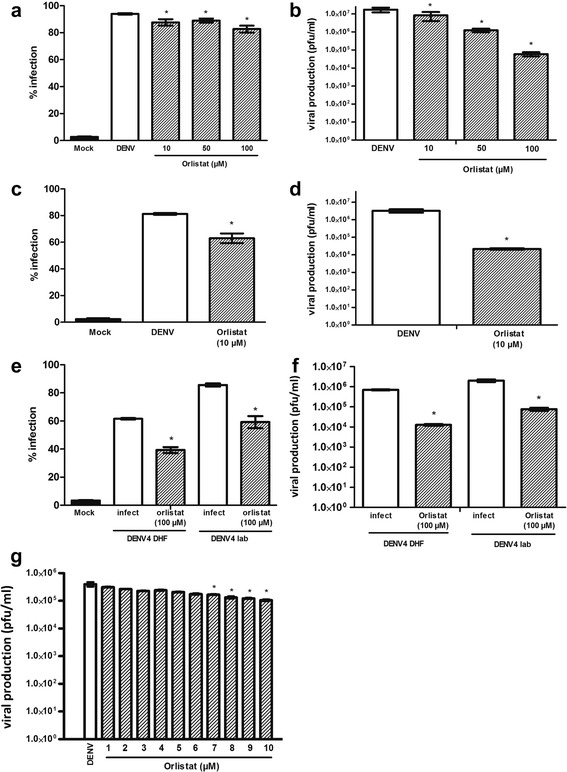
DENV 2 infection of HEK293T/17 cells treated with orlistat. HEK293T/17 cells were treated with the concentrations of orlistat as indicated or not treated (DENV) and subsequently infected with DENV 2 (**a**-**d**, **g**) or DENV-4DHF or DEN-4LAB (**e**,**f**). At 24 (**a**, **b**, **e**, **f**, **g**) or 36 h.p.i (**e**,**f**) the percentage infection and (**a**, **c**, **e**) and virus titer (**b**,**c**,**d**,**g**) were determined. Experiments were undertaken independently in triplicate, with duplicate plaque assay. Bars show mean +/−SD (*; *p* value <0.05)

HEK293T/17 cells were therefore incubated for 1 h in the presence or absence of the drug before infection with DENV 2 in the absence of the drug. Cells were cultured under standard conditions in the presence or absence of the compound as appropriate, and at 24 or 36 h post infection the percentage infection and virus titer were determined. These early time points were selected to avoid complications from the induction of apoptosis which occurs at later time points. Results at 24 h post infection (Fig. 7a) show that while the percentage of infected cells was not greatly reduced (albeit by a significant amount), the virus titer was significantly reduced by treatment with orlistat (Fig. 7b). At 36 h post infection, the percentage of cells infected was reduced slightly but significantly for orlistat treated cells (Fig. 7c) while virus titers were reduced by nearly 3Log10 for orlistat at 10 μM (Fig. 7d). Because of the noted morphological changes seen at higher concentrations of orlistat at 36 h, virus titers were not evaluated for these concentrations. The EC50 values for reduction of virus production for orlistat were 84.79 μM and 10.07 μM at 24 and 36 h respectively.

To determine if orlistat showed broad serotype and strain type activity, HEK293T/17 cells were again incubated for 1 h in the presence or absence of the 100 μM of orlistat before infection with a laboratory adapted DENV 4 (DENV-4LAB) or a low passage DENV 4 isolated from a dengue hemorrhagic fever patient (DENV-4DHF) as previously described in the absence of the drug. Cells were cultured under standard conditions in the presence or absence of 100 μM orlistat as appropriate, and at 24 h post infection the percentage infection and virus titer were determined. Results (Fig. 7e, f) showed a reduction in percentage infection for both viruses in the presence of the drug (Fig. 7e), and an approximately 2Log10 reduction in virus titers for the two viruses (Fig. 7f).

To determine the effect of the compound at lower concentrations, the effect of the compound on DENV 2 viral production was examined between 1 and 10 μM. Results (Fig. 7g) showed a consistent, dose dependent reduction in virus titer, with significant reductions in virus titer starting from 7 μM.

**Fig. 8:**
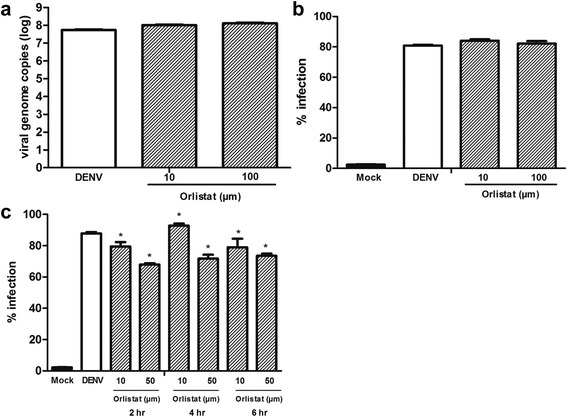
DENV 2 infection of HEK293T/17 cells pre-and post-treated with orlistat. **a**, **b** HEK293T/17 cells were not treated or treated with orlistat for 6 h before washing and subsequent infection with DENV 2. Cells were either (**a**) immediately harvested post infection and the viral genome copy number determined by qRT-PCR or (**b**) cultured for 24 h in the absence of the drug and percentage infection determined by flow cytometery. **c** HEK293T/17 cells were mock infected or infected with DENV 2 before culture under standard conditions either without further treatement (DENV) or with the addition of orlistat at the indicates times post-infection. At 24 h.p.i. cells were harvested for determination of percentage infection by flow cytometry. Experiments were undertaken independently in triplicate. Bars show mean +/−SD (*; *p* value <0.05)

To determine if the effect of orlistat was due to effects on viral entry, cells were not treated or pre-treated with orlistat for 6 h, washed and infected with DENV 2. In the first experiment, immediately after the infection period the cells were harvested and the level of the DENV viral genome established by qRT-PCR. Results (Fig. 8a) showed no difference in levels of viral genome between treated and non-treated cells, suggesting that orlistat does not exert its effect though altering viral uptake. In the second experiment cells were again pre-treated for 6 h with orlistat, washed and infected with DENV and then cultured under standard conditions without the drug for 24 h before determining the percentage infection. Results (Fig. 8b) showed that there was no difference in percentage infection when the cells had only been pre-treated.

To further confirm that orlistat was acting during infection and not at viral entry, cells were infected under standard conditions and orlistat added to the medium at 2, 4 and 6 h post infection. Significant differences in percentage infection were observed when the drug was added at all time points post infection (Fig. 8c).

### Effect of compounds on dengue protein expression

**Fig. 9:**
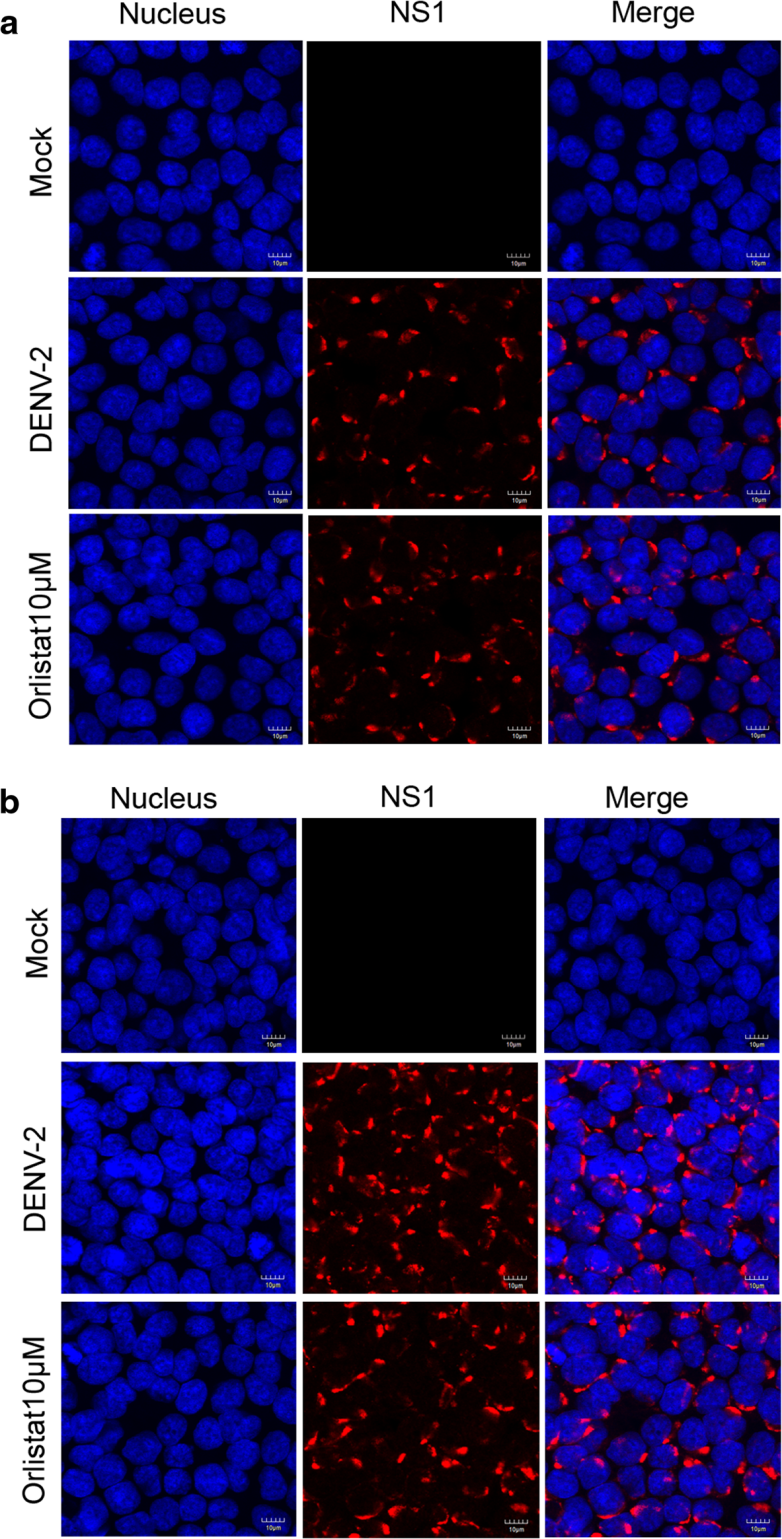
Effect of orlistat on DENV 2 NS1 expression. HEK293T/17 cells were pre-treated with 10 μM orlistat or not treated before mock infection or infection with DENV 2. At (**a**) 24 or (**b**) 36 h.p.i cells were fixed and stained to show the nucleus (*blue*) and DENV 2 NS1 protein (*red*). Cells were examined under an Olympus FluoView 1000 confocal microscope with 60× magnification. Representative, non-contrast adjusted merged images are shown

**Fig. 10:**
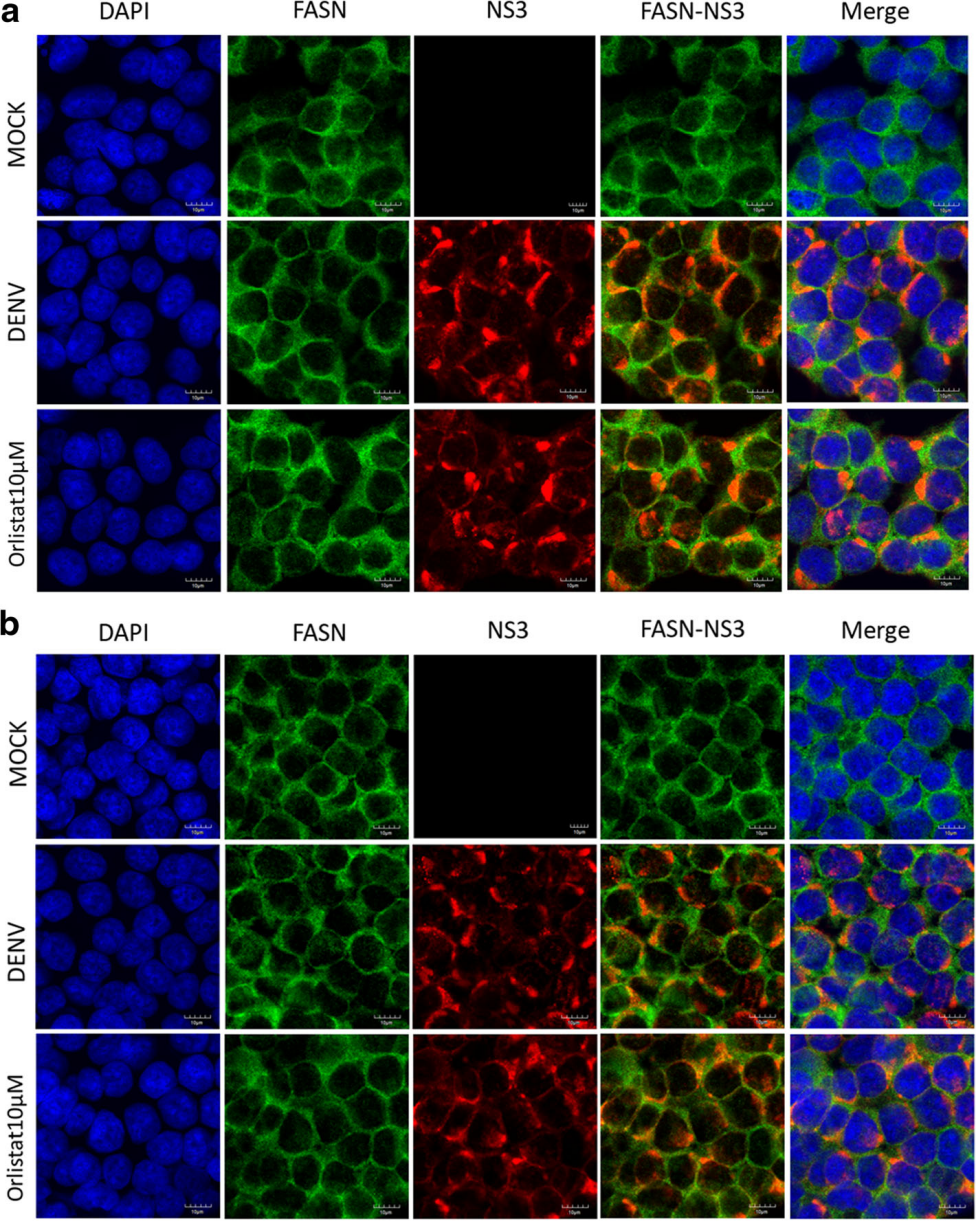
Double staining for FASN and NS3. HEK293T/17 cells were pre-treated with 10 μM orlistat or not treated before mock infection or infection with DENV 2. At (**a**) 24 or (**b**) 36 h.p.i cells were fixed and stained to show the FASN (*green*), DENV 2 NS3 protein (*red*) and nuclei (*blue*). Cells were examined under an Olympus FluoView 1000 confocal microscope with 60× magnification. Representative, non-contrast adjusted merged images are shown

To determine the effects of the compounds on DENV protein expression, HEK293T/17 cells grown on glass cover slips were infected with DENV 2 and treated with orlistat at a concentration of 10 μM. At 24 and 36 h post-infection, cells were observed under a confocal microscope for expression of DENV NS1 after staining with an anti-NS1 antibody. Results (Fig. 9) showed no observable reduction in NS1 signal with either compound at either time point. We additionally observed expression of DENV NS3 protein, and, as studies have shown that FASN is relocalized through interaction with NS3 and colocalizes with NS3 [25] we undertook a double staining to look at expression of both DENV NS3 and FASN. Results (Fig. 10) showed no observable reduction in DENV NS3 expression in the presence of the compounds, and, markedly, no significant colocalization between DENV NS3 and FASN was observed (Pearsons correlation coefficient 0.082 ± 0.03). We additionally tried co-immunoprecipitation experiments to confirm the interaction between NS3 and FASN, but despite repeated attempts, we were unable to do so (data not shown).

**Fig. 11:**
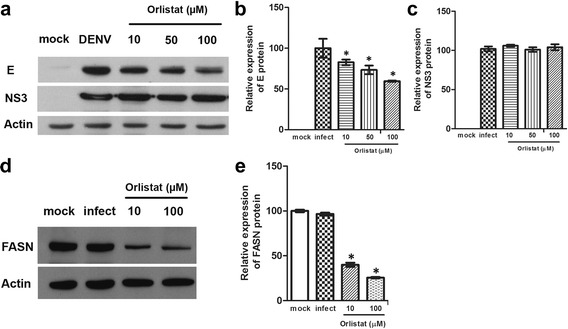
Western blot analysis of DENV 2 structural and non-structural proteins. HEK293T/17 cells were treated with the concentrations of orlistat as indicated or not treated (DENV) and subsequently mock infected (mock) or infected with DENV 2. At 24 h.p.i the expression of (**a**) DENV 2 E and NS3 proteins and (**d**) FASN was determined by western blotting. All filters were stripped and re-probed with actin as a loading control. Experiments were undertaken independently in triplicate and signals quantitated (**b**, **c**, **e**). Bars show mean +/−SD (*; *p* value <0.05)

To confirm the apparent lack of reduction of DENV protein expression under compound treatment, HEK293T/17 cells were again infected in the presence or absence of orlistat or mock infected, and expression of both structural (DENV E protein) and non-structural (DENV NS3) proteins as well as FASN were determined by western blotting in parallel with determining expression of actin. Results (Fig. 11) showed no change in levels of NS3 expression for either drug, while levels of E protein were reduced to about 50% at the highest level of orlistat used (100 μM). The results are consistent with the observations taken under confocal microscopy in which the significant decrease in virus output was not associated with a significant decrease in DENV NS3 protein expression. Interestingly, a significant reduction in FASN expression with DENV infection and orlistat treatment was seen even with treatment at 10 μM (Fig. 13).

## Discussion

Increasing attention is being focused on the role and involvement of lipids in DENV infection, and several studies undertaken on samples from DENV patients have shown that there are distinct changes in the lipidome of DENV infected patients [31, 32]. This is supported by studies that have shown lipid re-modeling in response to DENV infection in both mammalian [22, 25] and insect [17] cells. In mammalian cells it is believed that the main consequence of activation of autophagy in DENV infection [23, 24] is increased β-oxidation of fatty acids [22] and that lipid remodeling is achieved through the re-localization of FASN to the replication complex by interaction with DENV NS3 [25, 26].

While we and others [22] show a reduction of lipid droplets in response to DENV infection, other have reported an increase in the number of lipid droplets [33, 34]. This is reflected in the conflicting roles proposed for lipid droplets during DENV infection. While some authors propose that lipid droplets represent an “energy sink” that is tapped during viral replication [22], others propose that lipid droplets serve as a binding site for the DENV C protein during DENV assembly [35, 36]. Thus the involvement of lipid droplets during DENV infection requires further investigation.

We have shown that in mammalian cells there are marked changes in expression of genes involved with lipogenesis, lipolysis and fatty acid oxidation in response to DENV infection, with the most common profile being an early increase in expression, followed by a decrease in expression over time. While this study used a targeted pathway analysis, other studies using unbiased approaches have also identified lipid metabolism genes as being differentially regulated. Sessions and colleagues undertook a cross platform analysis of a number of studies and both FASN and DGAT1 were identified as being differentially regulated [37]. The alterations in lipid gene expression showed both cell type and virus strain variations, suggesting that there are differences in how different viruses re-model lipid metabolism. This is consistent with our previous observation that different DENV strains modulate the host cell proteome largely independently of virus serotype or passage history [27].

There was a large degree of discord between gene expression as evaluated at the level of the message and at the level of the protein. However, a number of large scale studies have clearly shown a high level of discordance between mRNA and protein levels [38–41]. This is attributed to a number of factors, particularly including protein stability and turnover. While our results showed large scale modulation of transcription, there was a much smaller effect on protein levels, suggesting a relatively large and stable protein pool associated with lipid metabolism. As such it is difficult to assess the biological relevance of the slight reduction in protein levels seen as a consequence of infection.

In contrast to the observations of others [25], we did not find a significant level of colocalization of FASN with NS3, and we were not able to confirm an interaction between these two proteins in immunoprecipitation experiments (data not shown). It is possible that this interaction is mediated by viral factors including virus strain. Importantly however, we have shown here that β-oxidation is not the only lipid metabolism pathway affected, but alterations in gene expression were additionally observed in lipogenesis and lipolysis.

Reduction of FASN through siRNA mediated knock down affected both virus titer as well as the percentage of infected cells, while orlistat mainly affected virus titer, with only minimal effects on the percentage of cells infected. This would suggest that knock down of FASN before infection results in a cellular environment unsuited to the establishment of infection, possibly by altering endosomal trafficking or another process required to establish the infection [17]. Another possibility is that lack of FASN may inhibit the formation of the DENV induced membranous structures [20] that serve to shield the DENV dsRNA from the host cell interferon response [21] again resulting in a more significant effect on DENV replication than simple biochemical inhibition of FASN. We note however that the knockdown of FASN was very transient which suggests that there is a high level of transcription of this gene, even under conditions of infection. However, confirming this would require absolute quantification of transcript levels rather than the comparative analysis undertaken here.

While siRNA mediated knock down of FASN had a larger effect on both virus production and percentage infection that treatment with orlistat, there is a less clear path to application in the treatment of DENV patients with siRNA based therapies than for the already licensed orlistat, and therefore evaluation of the effects of knock down of FASN by siRNA on DENV infection were less comprehensive than the studies undertaken with orlistat.

Treatment of cells with orlistat significantly reduced viral titer, albeit again with some strain related variation. Interestingly, while no significant reduction in NS3 levels was observed E protein expression was reduced to about 50% in orlistat treated cells. This suggests that the biochemical inhibition of FASN (as opposed to knock down) results in a reduction of the ability of viral particles to correctly form and be exported from the cells. This would support the contention of Kuhn and co-workers [25] that the major effect of lipid re-modeling in DENV infection is to influence membrane architecture to facilitate viral formation and egress. However, the reduction of E protein expression observed under orlistat treatment suggests that additional mechanisms are involved with this drug which may involve the specific clearance of E protein from the infected cell. Interestingly, a much greater reduction of FASN expression was seen in cells that were treated with orlistat and infected, as compared to cells that were only infected. This might indicate that under conditions of DENV infection there is a faster turnover of non-functional proteins.

Orlistat predominantly has activity against FASN expressed in the cells of the gut, and acts to reduce dietary fat uptake, hence its applications to the treatment of obesity.[42] However, some orlistat is available systemically [43, 44], suggesting possible application in anti-DENV therapy. The need for further exploration of orlistat as a potential anti-dengue therapy has been proposed [45].

However the EC value of orlistat is in the μMolar range, suggesting that other, more potent compounds targeting to FASN or other modulators of lipid metabolism [46] will be of better application. For example previous studies of the FASN inhibitor C75 have shown a CC_50_ value of >100 μM and an EC_50_ value of 5.7 μM [47] and an approximately 2Log10 reduction in viral titer [25].

## Conclusions

Increasing attention has been paid to the role of lipids and lipid metabolism during DENV infection [22, 25, 26, 31–34, 36, 45–48] and to methods of targeting components of this cellular process as an anti-DENV therapy. This study has confirmed that there is dysregulation of expression of lipid metabolism associated genes in DENV infection, although the effect on protein expression is markedly more muted, possibly as a reflection of protein turnover times. Interference with FASN, whether through knock down or chemical interference had a marked effect on viral output, although again there was some disconnect with the expression of non-structural proteins, suggesting that one of the main effects of alterations in lipid metabolism is on the ability of the virus particle to form as proposed by others [25]. Overall this work supports earlier studies and highlights the utility of further investigations on modulating lipid metabolism as a viable ant-DENV therapeutic approach.

## Additional files

Additional file 1: Table S1. List of gene specific primers (PDF 56 kb)
Additional file 2: Figure S1. Real-time PCR validation of siRNA mediated gene silencing of fatty acid synthase (FASN) gene. HEK293T/17 cells were not treated (mock) or treated with a siRNA control (GFP) or treated with one of four siRNAs directed to FASN (FASN1 to FASN 4). On days 1 to 5 post transfection the level of FASN transcript was determined by real time PCR. Normalization expression data relative to actin is shown. Bars show mean +/−SD. (a) 1 day post transfection, (b) 2 days post transfection, (c) 3 days post transfection, (d) 4 days post transfection and (e) 5 days post transfection. Bars show mean +/−SD (*; *p* value <0.05). **Figure S2**. Assessment of cell viability after siRNA transfection. HEK293T/17 cells were not treated (mock) or treated with a siRNA control (GFP) or treated with siRNAs directed to FASN (FASN1 and FASN 4). On day 2 post transfection cell viability was assessed by trypan blue staining and counting cells using a hemocytometer. Bars show mean +/−SD. **Figure S3**. Real-time PCR validation of siRNA mediated gene silencing of fatty acid synthase (FASN) gene. HEK293T/17 cells were not treated (mock) or treated with a siRNA control (GFP) or treated with one of four siRNAs directed to FASN (FASN1 to FASN 4). On day 2 post transfection (a) the level of FASN transcript was determined by real time PCR and (b) amplification product was run on an agarose gel and products visualized after ethidium bromide staining. Normalization expression data relative to actin is shown. Bars show mean +/−SD (*; *p* value <0.05). **Figure S4**. Western analysis of FASN expression after siRNA treatment. HEK293T/17 cells were not treated (mock) or treated with a siRNA control (GFP) or treated with one of four siRNAs directed to FASN (FASN1 to FASN 4). On days 1 to 4 post transfection the level of FASN protein was determined by western blot analysis. Normalization expression data relative to actin is shown. Bars show mean +/−SD (* *p* value <0.05; ** *p* value <0.01). **Figure S5**. Determination of orlistat cytotoxicity to HEK293T/17 cells. HEK293T/17 cells were incubated with different concentrations of orlistat or not treated (−) for (a) 24 h or (b) 36 h followed by MTT cell viability assays. Data is derived from 8 replicates. Treatment with 5% DMSO was used as a positive control. Bars show mean +/−SD (*; *p* value <0.05). **Figure S6**. The morphology of HEK293T/17 cells after orlistat treatment. HEK293T/17 cells were incubated with different concentrations of orlistat or not treated (mock) for (a) 24 h or (b) 36 h followed by observation under an inverted microscope. Magnification × 20. **Figure S7**. Evaluation of virucidal activity of orlistat. Stock DENV-2 was incubated with orlistat at concentrations of 1, 10, 20, 50 μM for 1 h and then used in the standard infection protocol. At 24 h.p.i (a) flow cytometry was performed to determine the percentage of infection and (b) supernatants were used to determine the virus titers. No deficit was observed in either percentage cell infection or virus titer. (PDF 2701 kb)

## Abbreviations

ACC1: Acetyl-CoA carboxylase alpha1
AOX: Fatty acyl CoA oxidase
ATGL: Adipose triglyceride lipase
CPT1: Carnitine palmitoyltransferase I
DENV: Dengue virus
DGAT1: Diglyceride acyltransferase 1
FASN: Fatty acid synthase gene
GFP: Green fluorescent protein
MCAD: Medium-chain acyl-CoA dehydrogenase
NS: Non-structural (protein)
PPARα: Peroxisome proliferator-activated receptor alpha
SCD1: Stearoyl-CoA desaturase-1
SREBP1c: Sterol regulatory element-binding protein-1c
