# Supplementary material for: Involvement of fatty acid synthase in dengue virus infection
Source: Virol J. 2017 Feb 13;14:28. doi: 10.1186/s12985-017-0685-9 (PMC5307738; doi:10.1186/s12985-017-0685-9)
Supplement: Additional file 1: Table S1. — List of gene specific primers (PDF 56 kb) [file 12985_2017_685_MOESM1_ESM.pdf]

Additional File 1

Involvement of fatty acid synthase in dengue virus infection

Natthida Tongluan[a], Suwipa Ramphan[a], Phitchayapak Wintachai[a], Janthima Jaresitthikunchai[b], Sarawut Khongwichit[a], Nitwara Wikan[a], Supoth Rajakam[a], Sutee Yoksan[a,c], Nuttaporn Wongsiriroj[a], Sittiruk Roytrakul[b] and Duncan R. Smith[a,c*]

[a]Institute of Molecular Biosciences, Mahidol University, Bangkok, 73170, Thailand
[b]National Center for Genetic Engineering and Biotechnology (BIOTEC), National Science and Technology Development Agency, Pathum Thani, 12120, Thailand
[c]Center for Emerging and Neglected Infectious Diseases, Mahidol University, Bangkok, 73170, Thailand

Table S1. List of gene specific primers

Gene name	Primer sequences	Tm
Acetyl-CoA carboxylase alpha1 (ACC1)	Forward: 5'-GCACCTGCTACTATTGCTACTC-3'<br>Reverse: 5'-CAGTCCCAGCACTCACATAAC-3'	62°C
Fatty acyl CoA oxidase (AOX)	Forward: 5'-CTTGCCCAGCTCATCACTAA-3'<br>Reverse: 5'-CCGATGTCACCAACGGTAAT-3'	62°C
Adipose triglyceride lipase (ATGL)	Forward: 5'-TGTCTGCAGCGGTTTCAT-3'<br>Reverse: 5'-CTCATAGAGTGGCAGGTTGTC-3'	62°C
Carnitine palmitoyltransferase I (CPT1)	Forward: 5'-CCTTCCCATTCGTAGCCTTT-3'<br>Reverse: 5'- CTTGCCCATGTCCTTGTAGT-3'	62°C
Diglyceride acyltransferase 1 (DGAT1)	Forward: 5'-CCTACCGCGATCTCTACTACTT-3'<br>Reverse: 5'-GGGTGAAGAACAGCATCTCAA-3'	62°C
Fatty acid synthase gene (FASN)	Forward: 5'-TGTCCTGGGAGGAGTGTAAA-3'<br>Reverse: 5'-CTGCTCCACGAACTCAAACA-3'	62°C
Medium-chain acyl-CoA dehydrogenase (MCAD)	Forward : 5'-GTAGCCCGTGAACACATTGA-3'<br>Reverse : 5'-TTCTGGAGCTGAAACAGTGG-3'	62°C
Peroxisome proliferator-activated receptor alpha (PPARα)	Forward: 5'-GGAGCTATGGTATGTGGTTCAG-3'<br>Reverse: 5'-CATCTGGTCTGTTGGTCTGTT-3'	62°C
Stearoyl-CoA desaturase-1 (SCD1)	Forward: 5'-CCTGCAGAATGGAGGAGATAAG-3'<br>Reverse: 5'-GCCTTCCTTATCCTTGTAGGTG-3'	62°C
Sterol regulatory element-binding protein-1c (SREBP1c)	Forward: 5'-CGCTCCTCCATCAATGACAA-3'<br>Reverse: 5'-GTGTTGCAGAAAGCGAATGTAG-3'	62°C
