# Supplementary material for: Involvement of fatty acid synthase in dengue virus infection
Source: Virol J. 2017 Feb 13;14:28. doi: 10.1186/s12985-017-0685-9 (PMC5307738; doi:10.1186/s12985-017-0685-9)
Supplement: Additional file 2: Figure S1. — Real-time PCR validation of siRNA mediated gene silencing of fatty acid synthase (FASN) gene. HEK293T/17 cells were not treated (mock) or treated with a siRNA control (GFP) or treated with one of four siRNAs directed to FASN (FASN1 to FASN 4). On days 1 to 5 post transfection the level of FASN transcript was determined by real time PCR. Normalization expression data relative to actin is shown. Bars show mean +/−SD. (a) 1 day post transfection, (b) 2 days post transfection, (c) 3 days post transfection, (d) 4 days post transfection and (e) 5 days post transfection. Bars show mean +/−SD (*; p value <0.05). Figure S2. Assessment of cell viability after siRNA transfection. HEK293T/17 cells were not treated (mock) or treated with a siRNA control (GFP) or treated with siRNAs directed to FASN (FASN1 and FASN 4). On day 2 post transfection cell viability was assessed by trypan blue staining and counting cells using a hemocytometer. Bars show mean +/−SD. Figure S3. Real-time PCR validation of siRNA mediated gene silencing of fatty acid synthase (FASN) gene. HEK293T/17 cells were not treated (mock) or treated with a siRNA control (GFP) or treated with one of four siRNAs directed to FASN (FASN1 to FASN 4). On day 2 post transfection (a) the level of FASN transcript was determined by real time PCR and (b) amplification product was run on an agarose gel and products visualized after ethidium bromide staining. Normalization expression data relative to actin is shown. Bars show mean +/−SD (*; p value <0.05). Figure S4. Western analysis of FASN expression after siRNA treatment. HEK293T/17 cells were not treated (mock) or treated with a siRNA control (GFP) or treated with one of four siRNAs directed to FASN (FASN1 to FASN 4). On days 1 to 4 post transfection the level of FASN protein was determined by western blot analysis. Normalization expression data relative to actin is shown. Bars show mean +/−SD (* p value <0.05; ** p value <0.01). Figure S5. Determination of orlistat cytotoxi [file 12985_2017_685_MOESM2_ESM.pdf]

Additional File 2

Involvement of fatty acid synthase in dengue virus infection

Natthida Tongluan[a], Suwipa Ramphan[a], Phitchayapak Wintachai[a], Janthima Jaresitthikunchai[b], Sarawut Khongwichit[a], Nitwara Wikan[a], Supoth Rajakam[a], Sutee Yoksan[a,c], Nuttaporn Wongsiriroj[a], Sittiruk Roytrakul[b] and Duncan R. Smith[a,c*]

[a]Institute of Molecular Biosciences, Mahidol University, Bangkok, 73170, Thailand

[b]National Center for Genetic Engineering and Biotechnology (BIOTEC), National Science and Technology Development Agency, Pathum Thani, 12120, Thailand

[c]Center for Emerging and Neglected Infectious Diseases, Mahidol University, Bangkok, 73170, Thailand

Figures S1 to S7

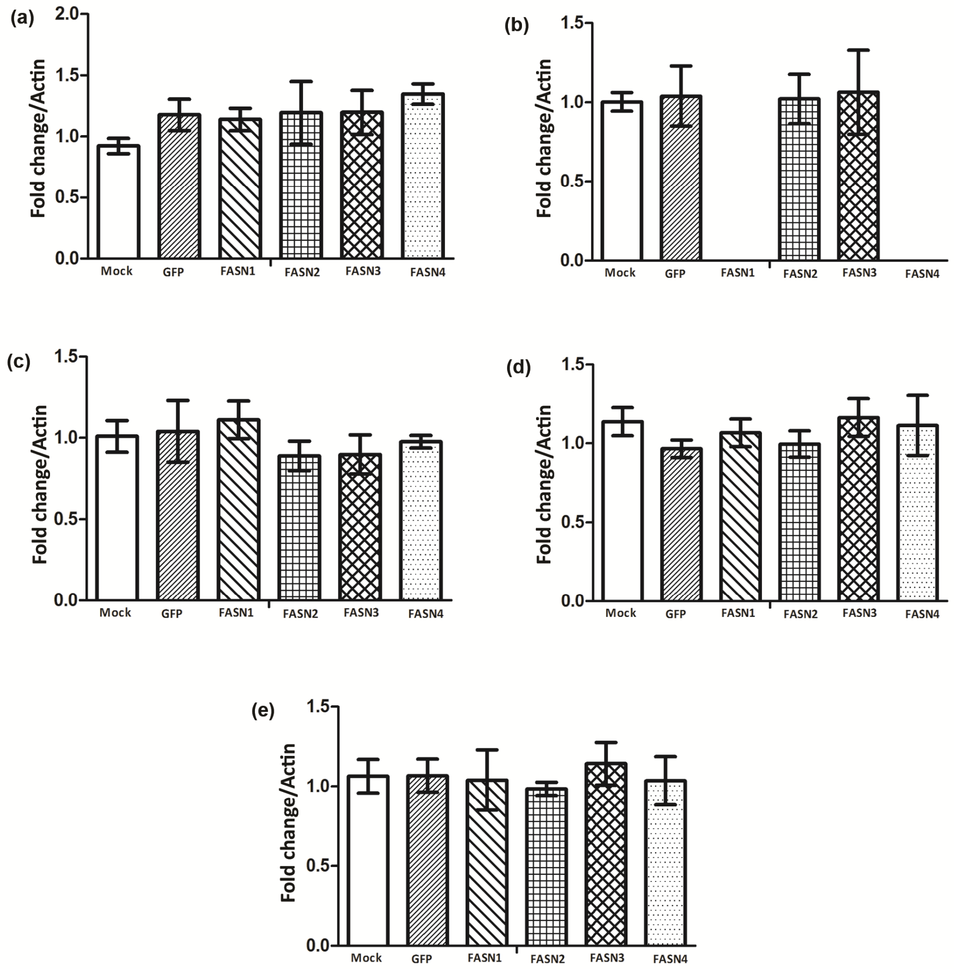

Figure S1. Real-time PCR validation of siRNA mediated gene silencing of fatty acid synthase (FASN) gene. HEK293T/17 cells were not treated (mock) or treated with a siRNA control (GFP) or treated with one of four siRNAs directed to FASN (FASN1 to FASN 4). On days 1 to 5 post transfection the level of FASN transcript was determined by real time PCR. Normalization expression data relative to actin is shown. Bars show mean +/- SD. (a) 1 day post transfection, (b) 2 days post transfection, (c) 3 days post transfection, (d) 4 days post transfection and (e) 5 days post transfection. Experiment was undertaken independently in triplicate. Bars show mean +/- SD (*; p value <0.05).

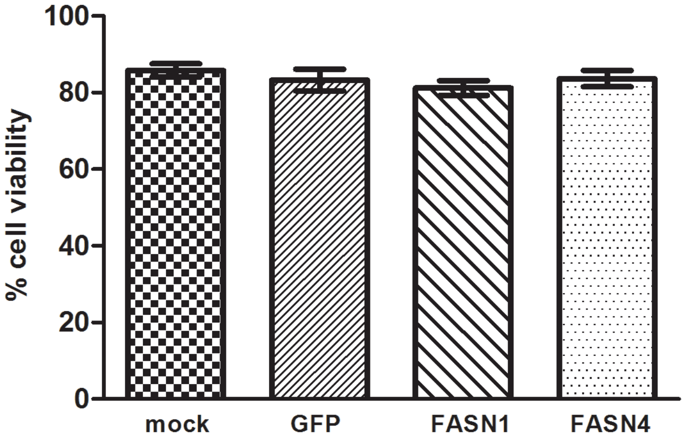

Figure S2. Assessment of cell viability after siRNA transfection. HEK293T/17 cells were not treated (mock) or treated with a siRNA control (GFP) or treated with siRNAs directed to FASN (FASN1 and FASN 4). On day 2 post transfection cell viability was assessed by trypan blue staining and counting cells using a hemocytometer. Experiment was undertaken independently in triplicate. Bars show mean +/- SD

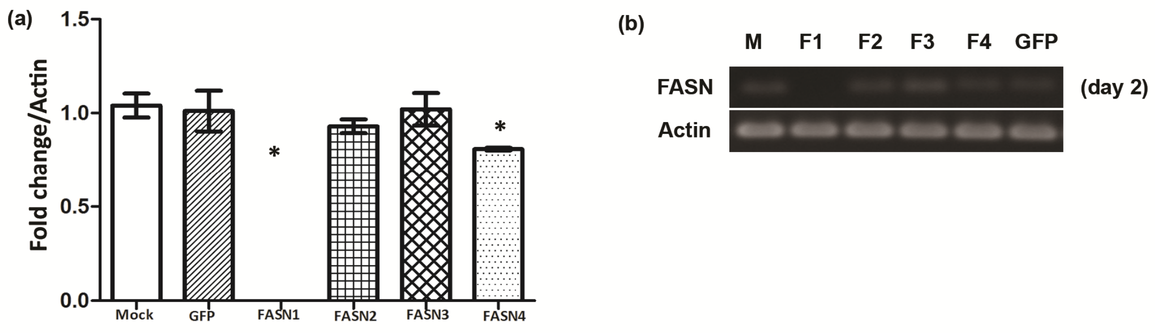

Figure S3. Real-time PCR validation of siRNA mediated gene silencing of fatty acid synthase (FASN) gene. HEK293T/17 cells were not treated (mock) or treated with a siRNA control (GFP) or treated with one of four siRNAs directed to FASN (FASN1 to FASN 4). On day 2 post transfection (a) the level of FASN transcript was determined by real time PCR and (b) amplification product was run on an agarose gel and products visualized after ethidium bromide staining. Normalization expression data relative to actin is shown. Experiment was undertaken independently in triplicate. Bars show mean +/- SD (*; *p* value <0.05).

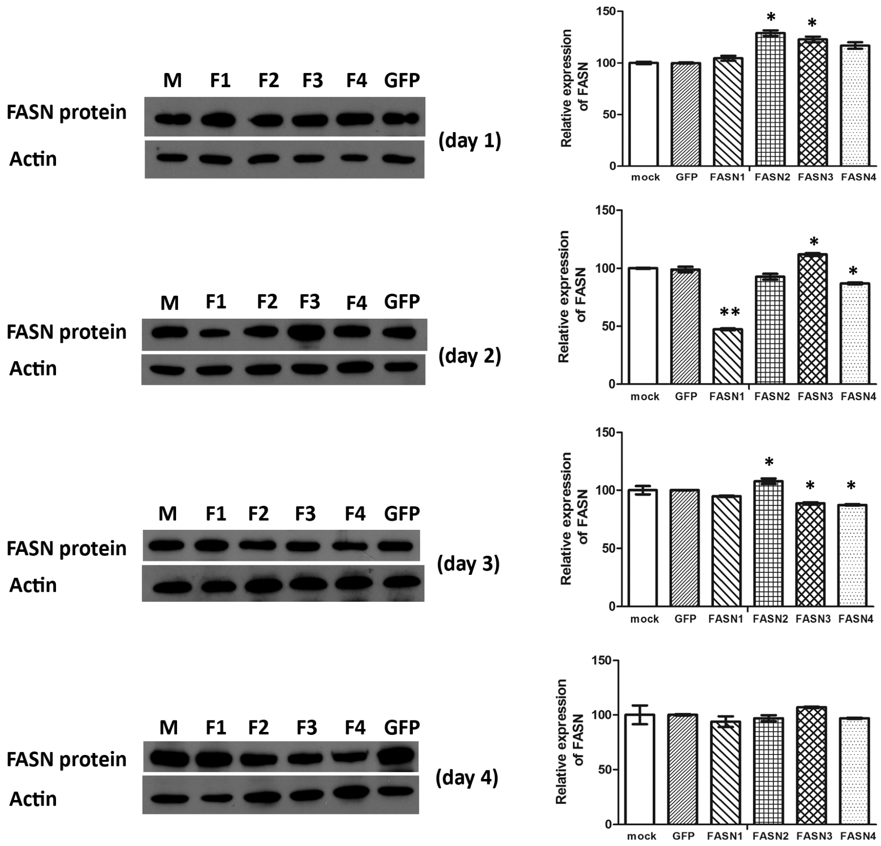

Figure S4. Western analysis of FASN expression after siRNA treatment.

HEK293T/17 cells were not treated (mock) or treated with a siRNA control (GFP) or treated with one of four siRNAs directed to FASN (FASN1 to FASN 4). On days 1 to 4 post transfection the level of FASN protein was determined by western blot analysis. Normalization expression data relative to actin is shown. Experiment was undertaken independently in triplicate, and representative Western blots are shown. Bars show mean +/- SD (* p value <0.05; ** p value <0.01).

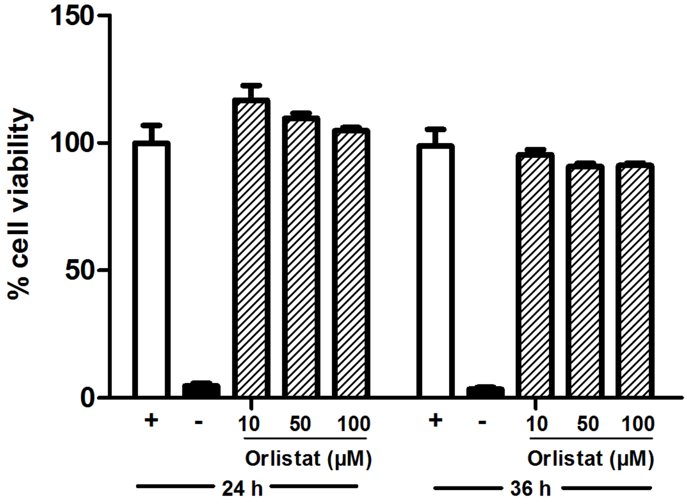

Figure S5. Determination of orlistat cytotoxicity to HEK293T/17 cells. HEK293T/17 cells were incubated with different concentrations of orlistat or not treated (-) for (a) 24 hours or (b) 36 hours followed by MTT cell viability assays. Data is derived from 8 replicates. Treatment with 5% DMSO was used as a positive control. Bars show mean +/- SD (*; p value <0.05).

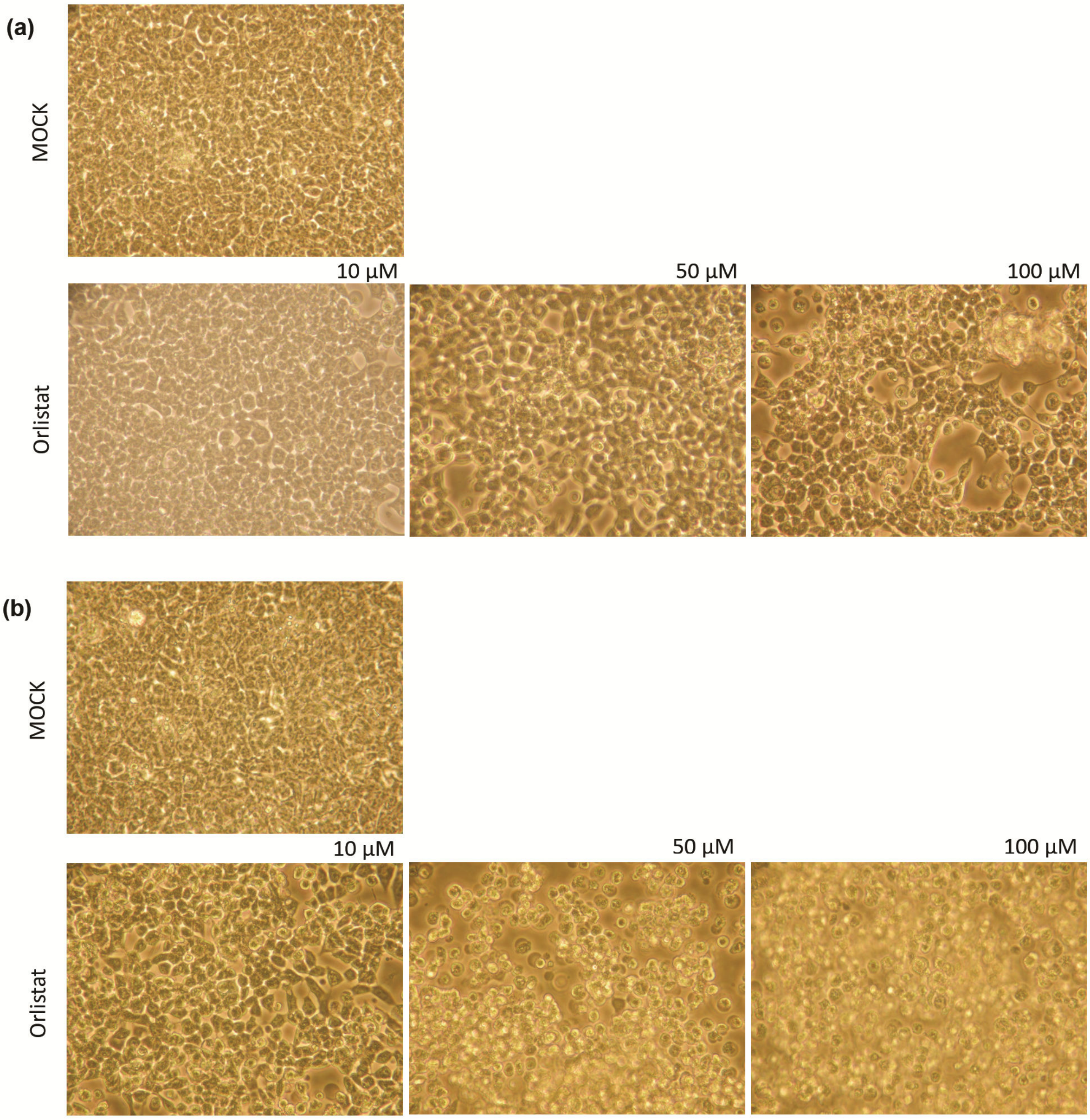

Figure S6. The morphology of HEK293T/17 cells after orlistat treatment.

HEK293T/17 cells were incubated with different concentrations of orlistat or not treated (mock) for (a) 24 hours or (b) 36 hours followed by observation under an inverted microscope. Magnification x 20.

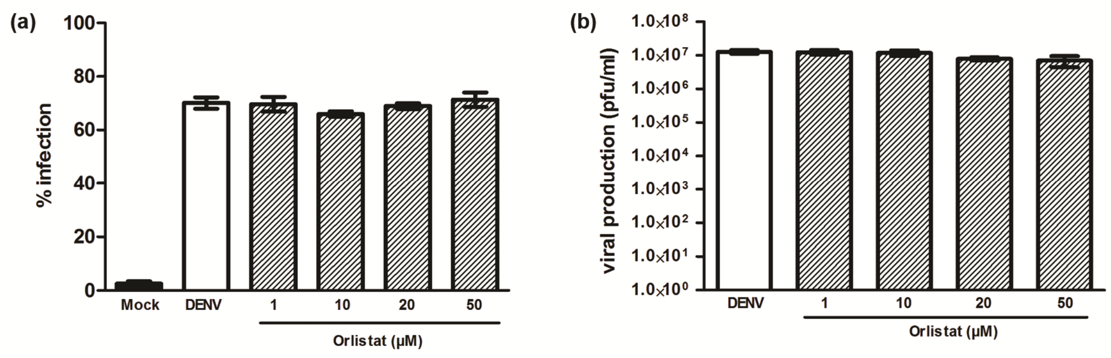

Figure S7. Evaluation of virucidal activity of orlistat. Stock DENV-2 was incubated with orlistat at concentrations of 1, 10, 20, 50 µM for 1 hr and then used in the standard infection protocol. At 24 h.p.i (a) flow cytometry was performed to determine the percentage of infection and (b) supernatants were used to determine the virus titers. No deficit was observed in either percentage cell infection or virus titer. Experiment was undertaken independently in triplicate with duplicate plaque assay.
